# Prevalence of Goiter and Associated Factors among Women of Reproductive Age Group in Demba Gofa Woreda, Gamo Gofa Zone, Southwest Ethiopia: A Community-Based Cross-Sectional Study

**DOI:** 10.1155/2020/5102329

**Published:** 2020-10-31

**Authors:** Befikaduwa Zekarias, Frehiwot Mesfin, Bezatu Mengiste, Adane Tesfaye, Lemma Getacher

**Affiliations:** ^1^Department of Public Health, College of Health and Medical Science, Mizan Tepi University, Tepi, Ethiopia; ^2^Department of Public Health, College of Health and Medical Science, Haramaya University, Haramaya, Ethiopia; ^3^Department of Nutrition, School of Public Health, College of Medicine and Health Science, Dilla University, Dilla, Ethiopia; ^4^Department of Public Health, College of Health Science, Institute of Medicine and Health Science, Debre Berhan University, Debre Berhan, Ethiopia

## Abstract

**Background:**

Iodine deficiency disorder is a major public health problem in Ethiopia that is more common in women of reproductive age. However, it is not well addressed and there is a lack of information on its prevalence and associated factors in women of reproductive age group. Therefore, the objective of this study was to assess goiter prevalence and associated factors among women of reproductive age in the Demba Gofa woreda, Gamo Gofa Zone, Southwest Ethiopia.

**Methods:**

A community-based cross-sectional study was used among 584 randomly selected women in the reproductive age group from February 05 to April 20, 2016. A simple random sampling technique was used to select the study kebeles, and a systematic random sampling technique was used to select the study samples. Data were collected through a pretested questionnaire, and the goiter examination was done clinically for each participant. The collected data were coded and entered into a computer for statistical analysis using EpiData version 3.2 and analyzed using SPSS version 20. Variables with a *P* value ≤0.25 in bivariate logistic regression analysis were entered into multivariate logistic regression analysis, and finally, variables with a *P* value <0.05 in multivariate logistic regression were considered significantly associated with the dependent variable.

**Results:**

The total goiter rate was 43%, 95% CI = 39.2–46.9. Cassava consumption (AOR: 2.02, 95% CI: 1.03–4), salt wash before use (AOR: 3.14, 95% CI: 1.1–11.3), salt use after >2 months of purchase (AOR: 11, 95% CI: 5–26), family history of goiter (AOR: 4.6, 95% CI: 1.4–15.8), and poor knowledge of iodized salt (AOR: 2.7, 95% CI: 1.4–5.5) were significant factors associated with goiter.

**Conclusion:**

Iodine deficiency was found to be severe in women of reproductive age in the study area. This showed that women of reproductive age, especially during pregnancy, are exposed to iodine deficiency and its adverse effects at delivery. Thus, they need urgent supplementation with iodine, improved access to foods rich in iodine, and intake of iodized salt. Additionally, health education should focus on the importance of iodized salt, the proper method of use, and the prevention of iodine deficiency, which are highly recommended to minimize the problem.

## 1. Background

Iodine, a trace element found in soil, is a key component of thyroid hormones involved in regulating the body's metabolic processes related to normal growth and development of most organs, especially the brain [1–3]. It is an essential micronutrient that our body cannot produce. Our body gets this micronutrient from the environment through food and drinks. In addition, it cannot be stored in the body for a long period of time; therefore, a daily supply is required [2].

Lack of adequate iodine in women prior to and during pregnancy could result in giving birth to children with irreversible brain damage. In addition, other complications during pregnancy are abortion, stillbirth, congenital defects, psychomotor defects, cretinism, mental retardation, paraplegia, deaf mutism, dwarfism, infant mortality, neonatal goiter, and hypothyroidism, which reduce school performance in children and cause goiters [3]. Iodine deficiency is the world's single most significant cause of preventable and irreversible brain damage and mental retardation [3, 4].

The most visible effect of iodine deficiency is goiter, a condition defined when each of the lateral lobes of the thyroid gland is larger than the terminal phalanxes of the thumb of the person examined, which is an indicator of chronic iodine deficiency [5]. Goiter can be used as a baseline assessment of a region's iodine status and as a sensitive long-term indicator for the success of an iodine programmer. Goiter due to iodine deficiency is measured through indictors, including urinary iodine concentration (UIC) and total goiter prevalence (TGP) and through the rates of coverage of adequately iodized salt (>15 ppm) in households. Goiter prevalence among school children and pregnant mothers is used to show the overall prevalence of goiter [6].

It has been reported that 2.2 billion people (38% of the world's population) live in areas with iodine deficiency and are at risk of its complications. Goiter due to iodine deficiency (ID) is the major public health problem in several areas of the world. Its prevalence in females in the reproductive age group is very high, ranging from 34.5% to 37% [3]. A study performed in India Kashmiri showed that the prevalence of grade one goiter was 12.68%, and the prevalence was high in the 20–24 years' age group [7]. Similarly, another study in Bahawalpur district in Pakistan showed that the prevalence of goiter in females was grade 0 = 35.7%, grade 1 = 42.85%, and grade 2 = 21.42%, and the prevalence decreased as age increased [8].

Iodine deficiency is as high as 42% in Africa. Approximately 350 million Africans remain under at risk of IDD, which is a quarter of the global burden of iodine deficiency (10). From 40 African countries that had urinary iodine concentration (UIC), fifteen countries (38%) were iodine-deficient, 22 countries (55%) had adequate iodine nutrition, and three countries (8%) had above the recommended intake. In Africa, the largest burden of ID, because of the large population size, was in Ethiopia, Sudan, Algeria, Morocco, Ghana, and Mozambique [9].

In Ethiopia, more than 35 million people are at risk of iodine deficiency, and approximately 28 million people have goiter. This makes it a major public health problem in the country [10]. Endemic and nonendemic areas have high goiter rates. From a global perspective, Ethiopia ranks 6th among 13 “make or break countries,” which significantly adds to the global high iodine deficiency disorders (IDD) burden. It is one of the nations with the lowest consumption of iodized salt (15%) and the highest goiter prevalence (36%) in Africa. Ethiopia has approximately 66 million persons “unprotected from iodine deficiency” [4].

Across the world, iodized salt and seafood are generally the major dietary sources of this nutrient [11]. The regular use of iodized salt constitutes simple prevention. “The amount of iodine needed over a lifetime is equivalent to a mere teaspoonful,” says Dr. Iqbal Kabir, a nutrition expert from UNICEF. “Prevention costs less than a cup of tea.” However, in Ethiopia, the preventive approach is so weak that only 15% of households use iodized salt. The percentage is higher in urban areas (23%) than in rural areas (14%). Even the coverage of iodized salt supplementation differs from region to region in Ethiopia; for example, the proportion of women living in a household with iodized salt is nearly three times higher in the Benshangul-Gumuz region (45%) than the national average (15%) [4, 12].

Regarding the causes of goiter, as different studies show, noniodized salt and insufficient iodine intake due to inadequate contents of iodine in seafood, dairy products, and iodized salt are the major causes of IDD. In addition, family history of goiter, depleted soil of iodine, crops grown on iodine-depleted soil, subsistence agriculture, exposure to goitrogenic food and substances, excessive cabbage consumption, poor time of salt storage (>2 months), poor knowledge and practice towards iodized salt and IDD prevention mechanisms, and sociodemographic factors such as age, occupation, income, family size, educational status of females and their husband, and occupation of women's and their husband are the major associated factors of goiter among the WRA group [5, 10, 12–17].

Although many studies show that IDD is the major public health problem in the country and that the problem is more common in females than in males, there is a dearth of information on the problem in women of child-bearing age. Most of the previous studies show only the prevalence of goiter among pregnant women, school children, and primary school girls. The previous studies are also inconsistent and do not indicate this problem in all WRA groups in a single study. In addition, these studies do not include the prevalence and factors of goiter among WRAs, including lactating mothers and adolescent girls, in a single study. Another important reason for this study is to see the spatial difference in the prevalence of goiter among these populations. Moreover, the study area, Demba Gofa woreda, is one of the endemic areas of goiter prevalence in the country. Therefore, the main objective of this study was to assess the prevalence and factors associated with goiter in women of reproductive age in Demba Gofa woreda.

## 2. Materials and Methods

### 2.1. Study Setting and Design

A community-based cross-sectional quantitative study method was applied from February 5–April 20, 2016, in Demba Gofa woreda. The study was carried out on four randomly selected kebeles in Demba Gofa woreda, which is one of the 15 woredas of the Gamo Gofa Zone in SNNPRS. The woreda is located 510 km south of Addis Ababa. The woreda is divided into 38 rural kebeles. The capital town of the woreda is Sawla. The study area has three agroclimatic zones: highland altitude/Dega/(7.8%), midaltitude/Woina Dega/(15.8%), and lowland altitude/Kola/(76.4%). The population of the woreda is 99,891 (male: 49446 and female: 50445 and from these 19819 are women in the reproductive age group). Maize and teff from cereals, sweet potatoes and cassava from roots, and *Moringa* (“Haleko”) and cabbage from vegetables were the major food types in this area. Cassava and cabbage are consumed in the great majority of households being processed, either alone or mixed with other cereal foods [8].

### 2.2. Study Population, Sample Size, and Sampling Procedures

Females in the child-bearing age group who were apparently healthy and willing to participate in the study were included in the study, while those who were severely ill to respond and had difficulty in speech or listing were excluded. To determine the sample sizes for the prevalence of goiter, single population proportion was used with the following assumptions to optimum sample size: *P* = prevalence of goiter to be 35.8% (95% CI 34.5–37.1) [5], *Z* = reliability coefficient = 1.96, *d* = 5% margin of error, design effect (DE) = 1.5, and nonresponse rate = 10%. Accordingly, the sample size was 584.

On the other hand, to determine the sample size for associated factors, a double population formula was used based on the following assumption: 95% confidence level, power 80%, ratio 1 : 1 and using the variable from a previous study, goiter: 73% (*P*1 = proportion for women with everyday cassava intake (exposed); 27% (*P*2 = proportion of women who never consume cassava) (unexposed), which gives the largest sample size among all other variables [2] using Epi Info version 7. Then, the calculated sample size was 44. Considering the 10% nonresponse and design effect, the sample size became 74. Therefore, comparing the two samples sizes, the maximum sample size (584) was taken as the final sample size of the study.

With regard to the sampling procedures, there were 38 kebeles in Demba Gofa woreda. Of these, Lamaya Tsala, Banda, Zanga, and Yala kebeles were randomly selected using lottery method. After calculating proportional sample size, the sample size was allocated proportionally to each kebele. Accordingly, the samples were 182 from Layma Thala, 113 from Banda, 186 from Zanga, and 103 from Yala kebeles. Then, the households from the selected kebeles were selected by systematic random sampling method (*N*/*n* = *n*th). The first household in each kebele was selected randomly by lottery method and the rest were every respected *n*th until the total sample size was achieved.

### 2.3. Data Collection Instruments and Procedures

A pretested and structured interviewer administered questionnaire that developed after reviewing different studies was used to collect the data [2–5, 7, 10, 12, 13, 16–18]. The questionnaire was initially prepared in English, translated into the local language (Amharic and Gofigna), and translated back to English to maintain its consistency. The questionnaires were grouped and arranged as sociodemographic characteristic of the respondents, quarries to assess contributing factors of goiter, quarries to assess knowledge and practice of the respondents towards utilization of iodized salt and IDD prevention, and evaluation and grading of goiter.

The data collectors of the study, three health officers and three girls who had completed the 10^th^ grade and who had previous data collection experience, were recruited. The health officers were trained by medical doctors on how to perform the standardized physical examination of goiters, and then they underwent goiter examination. The girls who completed the 10^th^ grade were trained by a principal investigator on how to interview the respondents for two days. Two supervisors, the medical doctors and the principal investigator, supervised the data collection. The collected data were checked by the supervisors and principal investigator on a daily basis for any incompleteness and possible corrections were made.

Regarding the data collection of the dietary characteristics of the respondents, there were questions used to assess the diets of the respondents. These questions assess the eating pattern of different food groups, such as meat/milk/fish, cassava, cabbage, and other important diets including the frequency of these foods in a day, week, and month.

The knowledge of respondents towards the prevention of IDD was assessed using six questions that assess the awareness of the participants of sources of information, causes of goiter, methods of prevention of goiter, utilization of iodized salt, and its storage.

Goiter examination was performed for all participants. To control errors and maximize the data quality, careful training and experienced observation were performed. To minimize interindividual variability using inspection and palpation, goiter grading was performed as per the recommendation of the 2014 WHO/UNICEF/ICCID. The classification scheme was as follows: grade 0: no goiter (neither visible nor palpable); grade 1: thyroid palpable but not visible; grade 2: thyroid visible with neck in normal position. The total goiter rate (TGR) was calculated as the sum of goiter grade 1 and grade 2. To minimize error during data entry, double data entry was implemented.

### 2.4. Data Processing and Analysis

The filled questionnaires were checked for completeness and entered into EpiData 3.1 statistical software and then exported to SPSS version 20 for further analysis. Descriptive statistics were used to describe the study population in relation to relevant variables. Both bivariate and multivariate logistic regression models were used to identify associated factors. To minimize factors in multivariate analysis, only variables that showed a *P* value ≤0.25 on the bivariate analysis were entered into the multiple binary logistic regression models.

To see the linear correlation among the independent variables, multicollinearity was checked using standard error (SE). Variables with a standard error of ≥2 were dropped from the multivariable analysis. The fitness of the model was tested by Hosmer–Lemeshow's goodness-of-fit test and Omnibus tests. The coefficient of fit was found to be insignificant with a large *P* value, and the Omnibus tests were significant. Odds ratios with 95% confidence intervals (CIs) were computed, and variables with *P* value less than 0.05 in multivariate analysis were considered statistically significant.

### 2.5. Ethics Approval and Consent to Participate

The study protocol was approved by Haramaya University, College of Health Ethical Review Board Committee (ERBC). The official letter of cooperation was written from Haramaya University, College of Health Science, to the study area. Before informed consent was obtained from each study participant, a clear description of the study title, purpose, procedures, durations, and possible risks and benefits of the study was explained. The rights and privacy of the study participants during the interview were guaranteed in advance and asked in a private room. For those whose age was less than 18 years, assent form from them and consent from their parent or husband was taken, and the responses of interviewees were kept confidential. Then, written and signed informed consent was obtained from each respondent before starting the interview. All questionnaires were coded with numbers to maintain the confidentiality of information gathered from each study participant throughout the study. Written informed consent was obtained from each participant.

## 3. Results

### 3.1. Sociodemographic Characteristics of Study Participants

Out of 584 respondents expected to participate in the study, 582 respondents were interviewed, resulting in a response rate of 99.6%. The mean age ±SD of the respondents was 30.49 ± 7.78 years. The majority of the respondents 500 (86%) were Gofa by ethnicity, half, 295 (50.5%), were protestant by religion, 193 (33.3%) were without formal education, 446 (76.4%) were married and 33,5 (57.5%) were housewives by occupation. Out of 446 married respondents, 37.4% and 58.5% of the respondents' husbands were without formal education and farmers, respectively. Approximately 56% of respondents had a family size greater than five (Table 1).

### 3.2. Dietary Characteristics and Familial History of Goiter of Respondents

The majority of the respondents, 462 (79.2%), eat fish/milk/meat; of these, 113 (24%) eat three times and above per week. More than three-fourths of the respondents, 444 (76%), consumed cassava, of whom 340 (76%) were eating three times and above per week. Of 485 (85%) cabbage consumers, more than four-fifths of 402 (83) consumed cabbage less than three times per week. More than three-fifths of respondents, 356 (61.2%), used coarse nonpacked salt, and only one-third (36.5%) of these respondents used packed iodized salt. Of those who used packed iodized salt, most (99.5%) used salt for less than six months. The majority of the respondents, 499 (85.7%), had no familial history of goiter (Table 2).

### 3.3. Knowledge of IDD of Respondents

In this study, more than three-quarters of respondents (71.6%) had poor knowledge of iodized salt and IDD.

### 3.4. Practice of Iodized Salt Utilization

Of the total respondents, most of them, 552 (95%), keep the salt in dry areas, but only one-third, 190 (32.5%), of respondents expose the salt to sun when the salt becomes humid. More than half of the respondents, 361 (62%), put the salt in a container having cover, but only 120 (19%) wash the salt before use. More than three-fourths of the participants, 399 (68.6%), used salt for ≤2 months once purchased. More than a half, 331 (56.9%), add salt at the end when cooking food, and only 160 (27.5%) have information about iodized salt. Of them, 97 (60.8%) receive the information from health professionals (Table 3).

### 3.5. Prevalence of Goiter among Women

**Figure 1:**
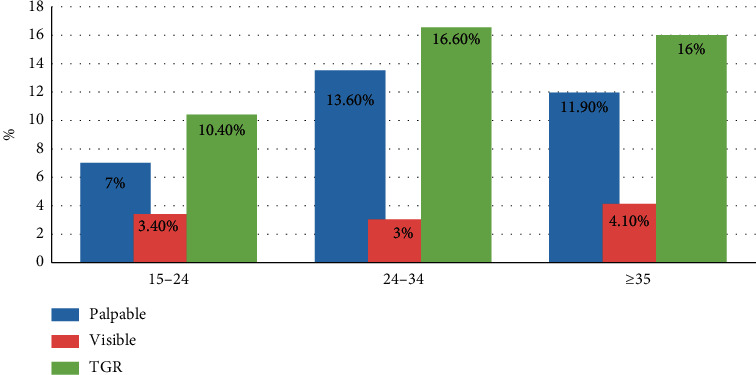
Prevalence of goiter in respondents by age category of women of reproductive age in Demba Gofa woreda, Southern Ethiopia, 2016.

The total goiter rate was 43.3% (95% CI = 39.2, 46.9), of which 32% was palpable and 11% was visible. The goiter rate was higher in the 24–34 years' age group (16.6%) (Figure 1).

### 3.6. Factors Associated with Goiter

In the bivariate logistic regression analysis, family size, fish/meat/milk consumption, cassava consumption, cabbage consumption frequency, family history of goiter, type of salt used, how the salt was stored, exposing the salt to sun, washing the salt before use, period of salt usage once purchased, the time to add the salt to the food, and knowledge towards IDD were significantly associated with goiter (Table 4). In the multivariate logistic regression analysis, income, cassava consumption, family history of goiter, salt wash before use, period of salt usage once purchased, and knowledge about IDD were significantly associated with goiter. Participants whose income was <500 ETB were four times (AOR: 4.2, 95% CI: 1.7–10) more likely to have goiter than those with income ≥999 (Table 4).

Respondents who had consumed cassava were 2 times (AOR: 2.02, 95% CI: 3.6–f4) more likely to have goiter than their counterparts. Family history of goiter also found a significant association with goiter. Respondents who had a family history of goiter were 4.6 times (AOR: 4.6, 95% CI: 1.4–15.8) more likely to have goiter than those had no family history of goiter. Participants who washed the salt before use were more than three times (AOR: 3.4. 95% CI: 1.1–11.3) more likely to have goiter than those who do not wash the salt.

The period of use salt at household once purchased was found to be a strong significant factor for goiter. Those who used the salt for more than two months were nearly twelve times (AOR: 11, 95% CI: 5–26) more likely to have goiter compared to their counterparts who used ≤2 months (Table 4).

Knowledge about IDDs was also found to have a significant association with goiter. Participants who had poor knowledge about IDD were nearly three times (AOR: 2.7, 95% CI: 1.4–5.5) more likely to have goiter than their counterparts (Table 4).

## 4. Discussion

The study revealed that the TGP was 43.3% (95% CI: 39.2, 46.9), of which 32% were palpable and 11% were visible goiters. Cassava consumption, family history of goiter, salt wash before use, period of salt usage once purchased, and knowledge towards IDD were significantly associated with goiter.

The prevalence of this study TGR was found to be higher than the national TGR, which was 35.8% (24.3% palpable and 11.5% visible) [5]. It was also higher than a study done in West Gojjam, which was 30.1% [13]. However, the current finding is below the regional state in Ethiopia TGR, which was 60% (43% palpable and 18% visible) [5], and a higher prevalence was found in the 25–34 years' age group. However, a study performed in Pakistan showed that the highest prevalence was found in the younger age group [18]. The increased rate of goiter in the current study might be due to Gamo Gofa being an endemic area for goiter, and the nutritional condition of the study population is quite different from other studies. The possible reason behind the discrepancy in age group might be related to the decrease in the prevalence of goiter as the age of these populations increases.

A longer (more than 2 months) storage time of salt at household once purchased was strongly associated with goiter. A study performed in Maychew district also showed that when the period of stay of salt at household increases once purchased, the adequacy of iodine in the salt will decrease [14]. A similar study conducted in Colombia showed that the effect of longer storage beyond 2 months aggravated losses of iodine from the salt [15]. Another study performed in Canada also strengthened this idea, with salt losses of 28%–51% and 35%–52% of its iodine content after three months and six months, respectively [19]. This is because when the salt is stored for a longer time and as iodine is an easily volatile element, iodine found in the salt will be easily loosened, and finally, the salt becomes inadequately iodized, which may result in goiter. On the other hand, due to different environmental conditions during storage and distribution, the quality of iodine may be affected.

Cassava consumption was identified to be significantly associated with goiter. Respondents who consumed cassava were more likely to have goiter than their counterparts who did not consume cassava. A similar study done at Sawla also showed that cassava consumption is a risk factor for goiter [20]. Likewise, a study conducted in five regions of Ethiopia in 2008 also showed that cassava consumption is a risk factor for IDD. In the two regions SNNP and Benshangul Gumuz, those who consumed cassava frequently were significantly affected by goiter than those who consume cassava rarely or never [16]. This is because cassava contains goitrogens such as thiocyanate and isothiocyanate that inhibit the uptake of iodine to thyroid follicular cells and block the thyroid peroxidase enzyme. In the presence of goitrogens, iodination of thyroglobulin protein will be affected, inducing poor thyroxine production.

Knowledge towards iodized salt and prevention of IDD was significantly associated with goiter. This study was consistent with the study done in Gondar [14], Mali [16], and Ghana [3]. This is because when the knowledge of participants towards the use of iodized salt is increased, they will have a good understanding of how to prevent IDD which was directly associated with the availability of adequate salt for all members of the household.

Salt wash before use was significantly associated with goiter. This finding is in agreement with the study done in Gondar town [14]. This is because as iodine is an easily volatile element, and when the salt is washed, the iodine in the salt will be easily loosened and result in inadequate of iodine in the salt; finally this results in goiter.

Family history of goiter was also found to be significantly associated with goiter. Respondents who had a family history were more likely to have goiter than those who had no family history of goiter [14]. This may be due to respondents having a family history of goiter and their families were living together and having similar dietary habits. Another reason may be that iodine deficiency is intergenerational, meaning that the deficiency passes through generations.

This finding will support the strategic objectives of the National Nutrition Program II (NNP-II) and Food and Nutrition Policy (FNP) in Ethiopia, which was launched to improve the nutritional status of women of reproductive age, including adolescent girls, pregnant women, and lactating mothers, through appropriate nutrition to alleviate the current burden of micronutrient deficiencies like IDD. In this study, consumption of cassava is significantly associated with goiter. It was also supported by the existing evidence. Therefore, based on this finding, avoiding and minimizing frequent consumption of cassava are recommended.

Regarding the limitations of the study, this study is limited because we did not use ultrasound which is the best way to assess goiter. Urinary iodine level is also better than assessing only by history and physical examination, as it may result in interobserver variation. However, the limitation was minimized by intensive training on goiter examination by medical doctors. The other limitation of this study is recall bias, which was minimized by probing the study participants.

## 5. Conclusion

Iodine deficiency was found to be a severe problem in women of reproductive age. Cassava consumption, family history of goiter, salt wash before use, period of salt usage once purchased, and knowledge of IDD were significantly associated with goiter.

Thus, urgent supplementation with iodine and improved access to foods rich in iodine and intake of iodized salt by nearby health facilities and health bureau are highly recommended. Health education on the importance of iodized salt and its proper usage and the effect of goitrogenic foods on the thyroid gland should be given by nearby health facilities and health bureau. Because IDD is the major public health problem in the country and still the prevalence is high in the study area, sectors of governmental, nongovernmental, and concerned bodies near the study area should collaborate to control or minimize the problem. Further studies should be conducted using larger sample sizes and biomarkers to verify factors influencing iodine deficiency disorder in Demba Gofa woreda communities.

## Figures and Tables

**Table 1:** Sociodemographic characteristics among women of reproductive age in Demba Gofa woreda, Southern Ethiopia, March 2016.

Variables	Frequency	Percentage (%)
*Age in years*		
15–24	150	25.8
25–34	244	41.9
≥35	188	32.3

*Religion*		
Protestant	295	50.7
Orthodox	215	36.9
Muslim	72	12.4

*Ethnicity*		
Gofa	500	86
Gamo	62	10.6
Others^*∗*^	20	3.4

*Marital status*		
Single	95	16.3
Married	446	76.6
Divorced	41	7.1

*Educational status of respondents*		
Without formal education	193	33.3
Read and write	115	19.7
Elementary school	202	34.6
High school and above	72	12.3

*Occupation of respondent*		
Housewife	335	57.5
Government employer	55	9.4
Self-employer	104	17.8
Student	88	15.2

*Educational status of husband*		
Illiterate	167	37.4
Read and write	51	11.4
Elementary school	120	26.9
High school and above	108	24.2

*Occupation of husband*		
Farmer	261	58.5
Merchant	90	20.2
Government employer	82	18.4
Others^*∗∗*^	11	2.5

*Family size*		
≤5	256	44.1
>5	326	55.9

^*∗*^Wolayta, Gurage, and Amhara. ^*∗∗*^Student and daily laborer.

**Table 2:** Dietary characteristics among women of reproductive age in Demba Gofa woreda, Southern Ethiopia, March 2016.

Variables	Total frequency	Percent (%)
*Eating fish/meat/milk*		
Yes	462	79.2
No	121	20.8

*Frequency of eating fish/meat/milk*		
<3 times/week	350	75.6
≥3 times/week	112	24.4

*Eating cassava*		
Yes	444	76.2
No	138	23.8

*Frequency of eating cassava*		
<3 times/week	340	76.6
≥3 times/week	104	23.4

*Eating cabbage*		
Yes	485	83
No	97	17

*Frequency of eating cabbage*		
<3 times/week	402	83
≥3 times/week	83	17

*Type of salt used*		
Packed iodized salt	226	38.8
Nonpacked (coarse salt)	356	61.2

*Familial history of goiter*		
Have	83	14.3
Do not have	499	85.7

**Table 3:** Knowledge and practice of iodized salt characteristics among women of reproductive age in Demba Gofa woreda, Southern Ethiopia, March 2016.

Variables	Frequency	Percentage (%)
*Place of keeping*		
Dry area	552	95
Moist and near to fire	30	5

*How to keep salt*		
Covered	361	62
Open	221	38

*Sun exposure*		
Yes	190	32.6
No	392	67.4

*Salt wash*		
Yes	114	19.6
No	468	80.4

*When to use salt*		
At the end	331	56.9
While cooking	251	43.1

*How long salt is kept*		
≤2 months	399	68.6
>2 months	183	31.4

*Information about iodized salt*		
Yes	160	27.5
No	422	72.5

*Knowledge towards prevention of goiter and IDD*		
Poor	414	71.8
Good	168	28.2

*Source of information about iodized salt*		
Health professionals	97	60.6
Radio	46	29
Others^*∗∗∗*^	17	10.4

^*∗∗∗*^Television and relativities.

**Table 4:** Results of multivariate logistic regression analysis of factors independently associated with goiter among women of reproductive age in Demba Gofa woreda, Southern Ethiopia, 2016.

Variables	Goiter		Crude OR (95% CI)	Adjusted OR (95% CI)
	Yes (%)	No (%)		
*Family size*				
<5	87 (33.8)	170 (76.2)	1.00	1.00
≥5	176 (54.6)	149 (45.4)	2.3 (1.6–3.2)	1.64 (0.65–2.24)

*Eating fish/meat/milk*				
Yes	144 (70.2)	205 (29.8)	1.00	1.00
No	38 (27.5)	93 (82.3)	3.3 (1.9–5.5)	1.1 (0.8–2.1)

*Eating cassava*				
Yes	225 (49.3)	219 (50.7)	2.7 (1.7–4)	**2.02 (1.03–4)** ^*∗*^
No	38 (27.5)	100 (72.5)	1.00	1.00

*Cabbage frequency*				
<3 times/week	168 (41.8)	234 (59.2)	1.00	1.00
≥3 times/week	64 (77)	19 (23)	4.7 (2.7–8.1)	0.96 (0.38–2.45)

*Family history of goiter*				
Have	73 (87.9)	10 (12.1	11.8 (5.9–23.5)	**4.6 (1.4–15.8)** ^*∗*^
Do not have	190 (38.1)	309 (61.9)	1.00	1.00

*Type of salt*				
Packed iodized	70 (30.9)	156 (69.1)	1.00	1.00
Nonpacked coarse	193 (54.2)	163 (45.8)	2.6 (1.85–3.75)	1.6 (0.8–3.2)

*How do you keep salt?*				
Closed container	99 (27.4)	262 (72.6)	1.00	1.00
Open container	164 (74.2)	57 (25.8)	7.6 (5.2–11.1)	0.5 (0.3–1.1)

*Sun exposure of salt*				
Yes	158 (83.2)	32 (16.8)	13.5 (8.7–20.9)	1.2 (0.5–2.80
No	105 (26.8)	287 (73.2)	1.00	1.00

*Wash salt*				
Yes	108 (94.7)	6 (5.3)	36 (15.6–84.6)	**3.4 (1.1–11.3)** ^*∗*^
No	155 (31.9)	313 (68.1)	1.00	1.00

*How long salt is used once purchased*				
≤2 months	91 (22.8)	308 (87.2)	1.00	1.00
>2 months	172 (93.9)	11 (6.1)	53 (27.5–102)	**11 (5–26)** ^*∗∗*^

*When to use salt in cooking food*				
At the end	78 (23.6)	253 (76.4)	1.00	1.00
While cooking	185 (81)	43 (19)	9.1 (6.2–13.3)	1.32 (0.8–2.12)

*Knowledge towards IDD*				
Good	204 (49.3)	210 (50.7)	1.00	1.00
Poor	59 (64.9)	109 (35.1)	1.79 (1.23–2.6)	**2.7 (1.4–5.5)** ^*∗*^

## Data Availability

The data used to support the findings of this study are available from Mrs. Befikaduwa Zekarias upon reasonable request.
